# Impact of Factor Xa inhibitors on cardiovascular events in older patients with nonvalvular atrial fibrillation

**DOI:** 10.18632/aging.206238

**Published:** 2025-04-10

**Authors:** Masahiko Takahashi, Takeshi Morimoto, Ryu Tsushima, Yuya Sudo, Ai Sakamoto, Masahiro Sogo, Masatomo Ozaki, Keisuke Okawa

**Affiliations:** 1 Department of Cardiovascular Medicine, Kagawa Prefectural Central Hospital, Takamatsu, Kagawa 760-8557, Japan; 2 Department of Clinical Epidemiology, Hyogo Medical University, Nishinomiya, Hyogo 663-8501, Japan

**Keywords:** Factor Xa inhibitor, atrial fibrillation, older patient, cardiovascular events

## Abstract

Background: Experimental studies have reported that Factor Xa inhibitors (Xa-Is) have positive effects on cardiac muscles and blood vessels via protease-activated receptor 2 inhibition, suggesting the preventive effects of Xa-Is on cardiovascular events. However, the clinical impact of Xa-Is on cardiovascular disease is unknown.

Objectives: This study aimed to investigate the incidence of cardiovascular events among older patients with nonvalvular atrial fibrillation (NVAF) taking Xa-Is compared with those taking non-Xa-Is.

Methods: We conducted a single-center historical cohort study of consecutive patients with NVAF who were aged ≥80 years and used oral anticoagulants. Xa-Is included rivaroxaban, apixaban, and edoxaban, and non-Xa-Is included dabigatran and warfarin. The outcome of cardiovascular events was defined as a composite outcome of congestive heart failure, arteriosclerotic disease, and cardiovascular death. We compared the 5-year incidence of cardiovascular events between patients taking Xa-Is and those taking non-Xa-Is.

Results: Of 1705 patients aged ≥80 years who were diagnosed with AF, 1092 patients with NVAF were enrolled. Propensity score matching provided 445 patients in each group. The risks of cardiovascular events, congestive heart failure, arteriosclerotic disease, and cardiovascular death were significantly lower in the Xa-I group than in the non-Xa-I group (hazard ratio [95% confidence interval]: 0.43 [0.30–0.61], 0.44 [0.29–0.66], 0.47 [0.22–1.04], and 0.41 [0.23–0.75], respectively).

Conclusions: Among patients with NVAF who were aged ≥80 years, the incidence of cardiovascular events was lower in the Xa-I users than in the non-Xa-I users.

## INTRODUCTION

Anticoagulation therapy is crucial among older patients with atrial fibrillation (AF) because the incidence of thromboembolisms increases with age [1, 2]. Similarly, older patients, especially those with AF, often have numerous cardiovascular events, such as heart failure and coronary artery disease [3–5].

Currently, direct oral anticoagulants (DOACs) have been established as standard anticoagulation drugs for all generations of patients. Studies have reported that the incidences of both thromboembolisms and major bleeding were lower in patients taking DOACs than in those taking warfarin [6], even among very old patients with nonvalvular AF (NVAF) [7].

DOACs include dabigatran as a direct thrombin inhibitor, and rivaroxaban, apixaban, and edoxaban as Factor Xa inhibitors (Xa-Is). A recent study among patients with NVAF and diabetes mellitus who were aged ≥65 years demonstrated that the incidence of hospitalizations for heart failure was lower in those taking DOACs than in those taking warfarin [8], suggesting that DOACs might have benefits beyond their anticoagulation effect. However, no clinical study has focused on the impact of Xa-Is on cardiovascular events. Recently, several experimental studies have suggested positive effects of Xa-Is on the cardiovascular system, other than anticoagulation, such as the prevention of the cardiac remodeling and atherosclerosis progression [9–13].

We hypothesized that Xa-Is have preventive effects on cardiovascular events in older patients. Thus, we investigated cardiovascular events, including heart failure, atherosclerotic disease, and cardiovascular death, in patients with NVAF who were aged ≥80 years and received anticoagulation therapy. Furthermore, we compared the 5-year incidence of cardiovascular events between patients taking Xa-Is and those taking non-Xa-Is.

## RESULTS

### Baseline characteristics

**Figure 1:**
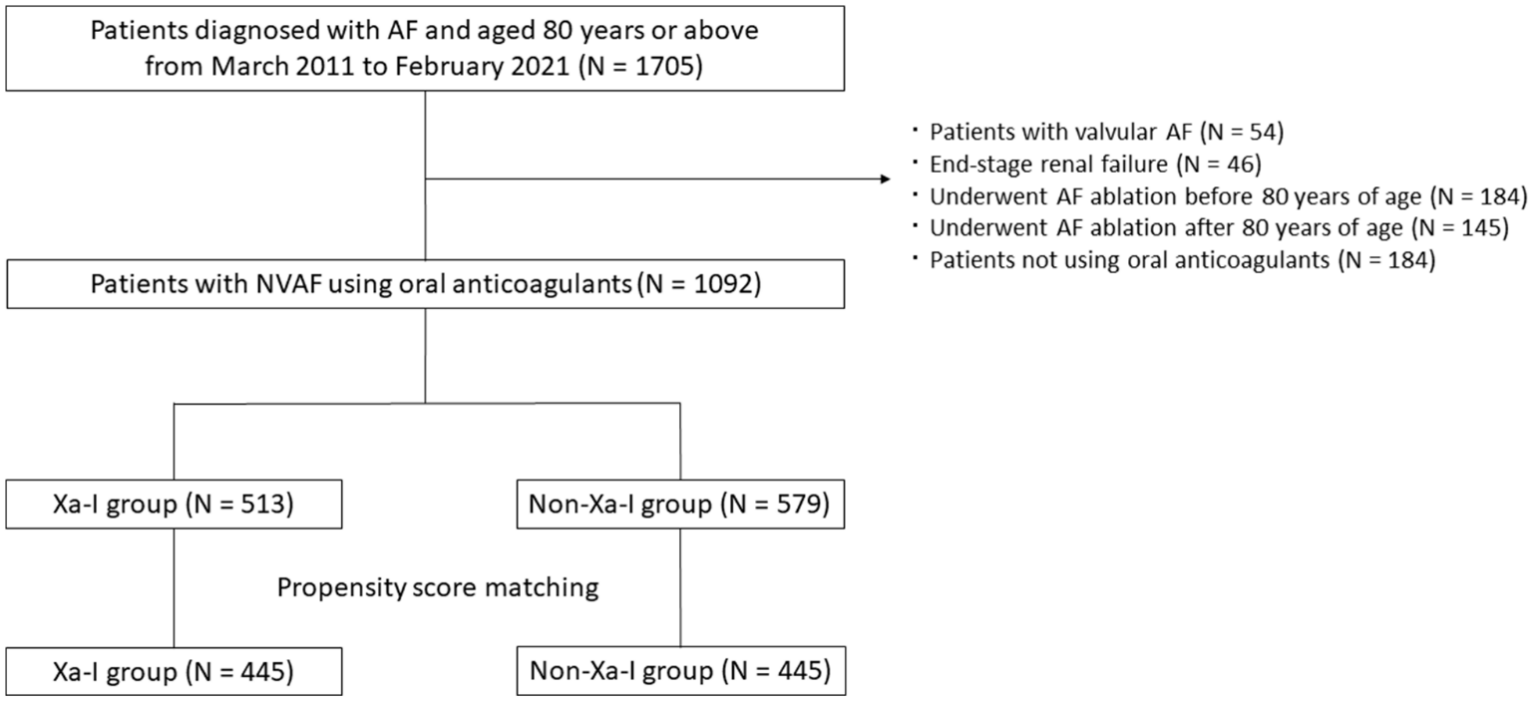
**Flowchart of the study procedure.** A total of 1705 patients aged ≥80 years were diagnosed with AF. Ultimately, 1092 patients with NVAF (Xa-I group, n = 513; non-Xa-I group, n = 579) were included in this study as the total original cohort. After propensity score matching, 445 patients were extracted from both groups. AF, atrial fibrillation; NVAF, nonvalvular AF; Xa-I, Factor Xa inhibitor.

Among 1705 patients aged ≥80 years who were diagnosed with AF, we excluded 54 with valvular AF, 46 with end-stage renal failure, 184 who underwent AF ablation before 80 years of age, 145 who underwent AF ablation after 80 years of age, and 184 who were not using oral anticoagulants (OACs). The reasons for the nonuse of OACs were based on the decisions of physicians, mainly considering the bleeding risk of patients. Among the patients not using OACs, 95% (n = 175) had at least one bleeding risk factor and 77% (n = 141) had more than two bleeding risk factors other than age. Consequently, 1092 patients with NVAF (Xa-I group, n = 513; non-Xa-I group, n = 579) were included in this study as a crude cohort (Figure 1).

The median observation period was 962 days (interquartile ranges [IQR]: 450, 1563) in the Xa-I group and 1180 days (IQR: 463, 1960) in the non-Xa-I group (P < 0.001). The distribution of Xa-Is was as follows: rivaroxaban, 18% (n = 95); apixaban, 59% (n = 302); and edoxaban, 23% (n = 116). The distribution of non-Xa-Is was as follows: dabigatran, 16% (n = 95) and warfarin, 84% (n = 484). In the Xa-I group, 87% (n = 445) of patients used low-dose Xa-Is. Among them, 77% (n = 344) of patients met the dose reduction criteria, and the remaining 23% (n = 99) of patients who were prescribed a low dose had at least one bleeding risk factor other than age. In the non-Xa-I group, dabigatran was used in the low-dose setting in 99% (n = 94) of patients who met the dose reduction criteria. Regarding warfarin control, in 87% (n = 428) of patients who could be evaluated for the time in a therapeutic range (TTR), the median TTR was 61% (IQR: 36, 87). The median age, proportions of female patients and patients with a history of heart failure, prevalence of hypertension, and β-blockers use were significantly higher in the Xa-I group than in the non-Xa-I group. The proportions of patients with a history of thromboembolisms were significantly lower in the Xa-I group than in the non-Xa-I group (Table 1).

**Table 1:** Baseline characteristics (total original cohort).

	**Xa-I group** **(N = 513)**	**Non-Xa-I group** **(N = 579)**	**P-value**
Follow-up period (days), median (IQR)	962 (450, 1563)	1180 (463, 1960)	< 0.001
Age (years), median (IQR)	84 (81, 87)	83 (80, 86)	0.003
Female sex, N (%)	272 (53)	265 (46)	0.017
Body weight (kg), median (IQR)	53 (46, 60)	54 (45, 61)	0.74
eGFR (mL/min/1.73 m^2^), median (IQR)	54 (44, 66)	55 (42, 68)	0.30
Persistent AF, N (%)	298 (58)	344 (59)	0.66
Hypertension, N (%)	378 (74)	374 (65)	0.001
Diabetes mellitus, N (%)	88 (17)	114 (20)	0.28
History of heart failure, N (%)	93 (18)	50 (9)	< 0.001
History of a pacemaker implantation, N (%)	58 (11)	70 (12)	0.69
History of arteriosclerotic disease, N (%)	133 (26)	133 (23)	0.26
History of a thromboembolism, N (%)	109 (21)	163 (28)	0.009
History of major bleeding, N (%)	26 (5)	40 (7)	0.20
Medications			
ACEI/ARB, N (%)	236 (46)	280 (48)	0.44
β-blocker, N (%)	262 (51)	216 (37)	< 0.001
Ca blocker, N (%)	248 (48)	249 (43)	0.08
Statin, N (%)	134 (26)	132 (23)	0.20

Values are presented as the median (IQR) in accordance with the distribution and number of patients (%).

Abbreviations: ACE-I, angiotensin-converting enzyme inhibitor; AF, atrial fibrillation; ARB, angiotensin receptor blocker; Ca blocker, calcium channel blocker; eGFR, estimated glomerular filtration rate; IQR, interquartile range; SD, standard deviation; Xa-I, Factor Xa inhibitor.

Within the observation period, 4.5% (n = 49) of patients (22 of 513 in the Xa-I group and 27 of 579 in the non-Xa-I group) were lost to follow-up. The rate of discontinuation of OACs was 3.8% (n = 41) (22 of 513 in the Xa-I group and 19 of 579 in the non-Xa-I group). The reasons for discontinuing OACs were minor bleeding (n = 11), aging (n = 2), renal function decline (n = 2), poor adherence or warfarin control (n = 3), an invasive procedure (n = 7), and unknown (n = 16).

After propensity score matching, 445 patients were selected from each group, and the baseline characteristics were well matched between the groups (Table 2).

**Table 2:** Baseline characteristics (propensity score-matched cohort).

	**Xa-I group** **(N = 445)**	**Non-Xa-I group** **(N = 445)**	**P-value**
Follow-up period (days), median (IQR)	998 (452, 1566)	1092 (436, 1903)	0.50
Age (years), median (IQR)	83 (81, 87)	83 (80, 86)	0.38
Female sex, N (%)	221 (50)	226 (51)	1.00
Body weight (kg), median (IQR)	53 (46, 60)	53 (45, 60)	0.89
eGFR (mL/min/1.73 m^2^), median (IQR)	55 (44, 66)	55 (41, 68)	0.95
Persistent AF, N (%)	261 (59)	256 (58)	0.79
Hypertension, N (%)	321 (72)	304 (68)	0.24
Diabetes mellitus, N (%)	80 (18)	87 (20)	0.61
History of heart failure, N (%)	41 (10)	49 (7)	0.43
History of a pacemaker implantation, N (%)	54 (12)	59 (13)	0.68
History of arteriosclerotic disease, N (%)	104 (23)	109 (24)	0.75
History of a thromboembolism, N (%)	101 (23)	101 (23)	1.00
History of major bleeding, N (%)	26 (6)	26 (6)	1.00
Medications			
ACE-I/ARB, N (%)	198 (44)	228 (51)	0.052
β-blocker, N (%)	204 (46)	203 (46)	0.96
Ca blocker, N (%)	211 (47)	195 (44)	0.31
Statin, N (%)	107 (24)	111 (25)	0.82

Values are presented as the median (IQR) in accordance with the distribution and number of patients (%).

Abbreviations: ACE-I, angiotensin-converting enzyme inhibitor; AF, atrial fibrillation; ARB, angiotensin receptor blocker; Ca blocker, calcium channel blocker; eGFR, estimated glomerular filtration rate; IQR, interquartile range; SD, standard deviation; Xa-I, Factor Xa inhibitor.

### Outcomes

**Figure 2:**
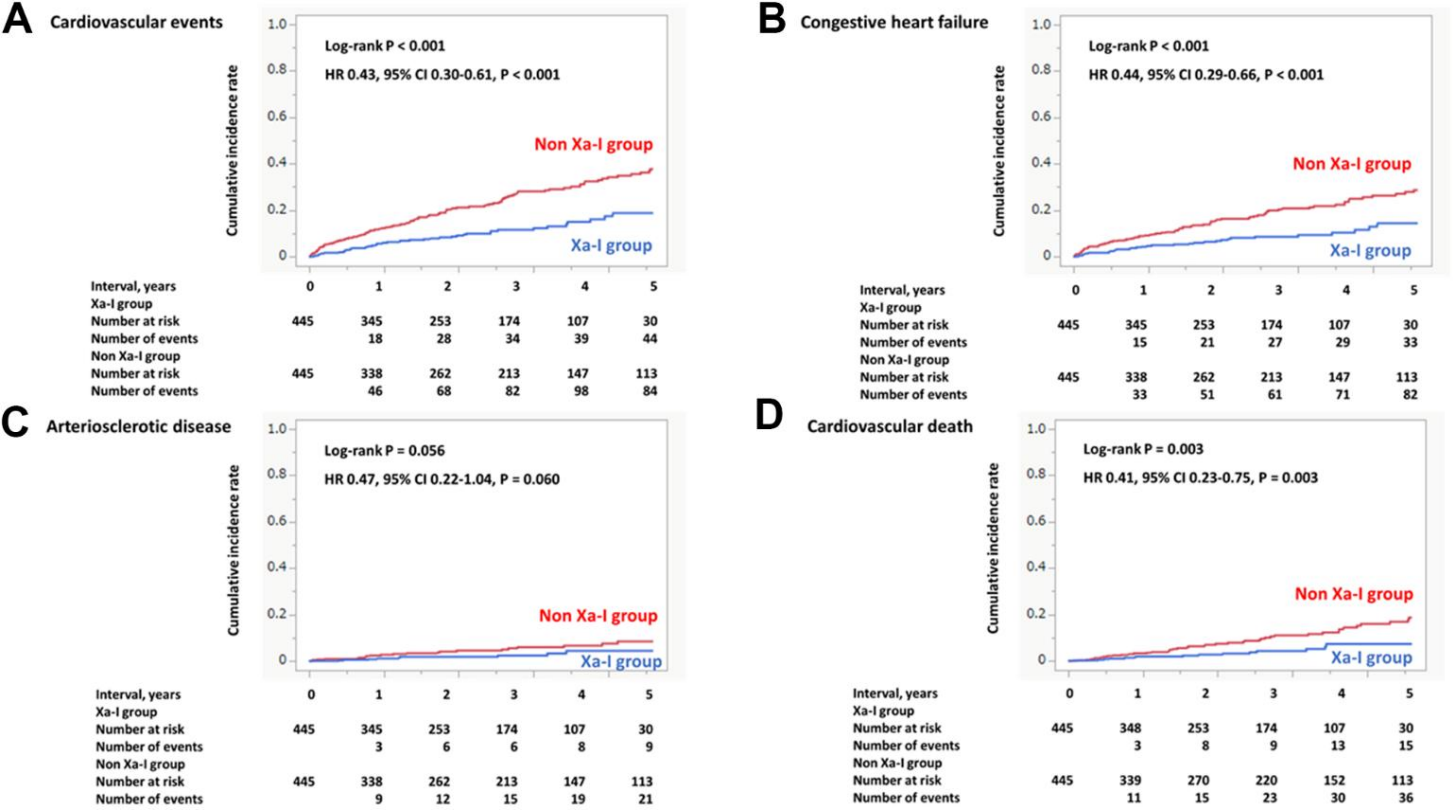
**Incidence of primary and secondary outcomes (propensity score-matched cohort).** (**A**) Cardiovascular events. (**B**) Congestive heart failure. (**C**) Arteriosclerotic disease. (**D**) Cardiovascular death. The incidence of composite cardiovascular events, arteriosclerotic disease, congestive heart failure, and cardiovascular death within 5 years was significantly and numerically lower in the Xa-I group than in the non-Xa-I group. CI, confidence interval; HR, hazard ratio; Xa-I, Factor Xa inhibitor.

**Figure 3:**
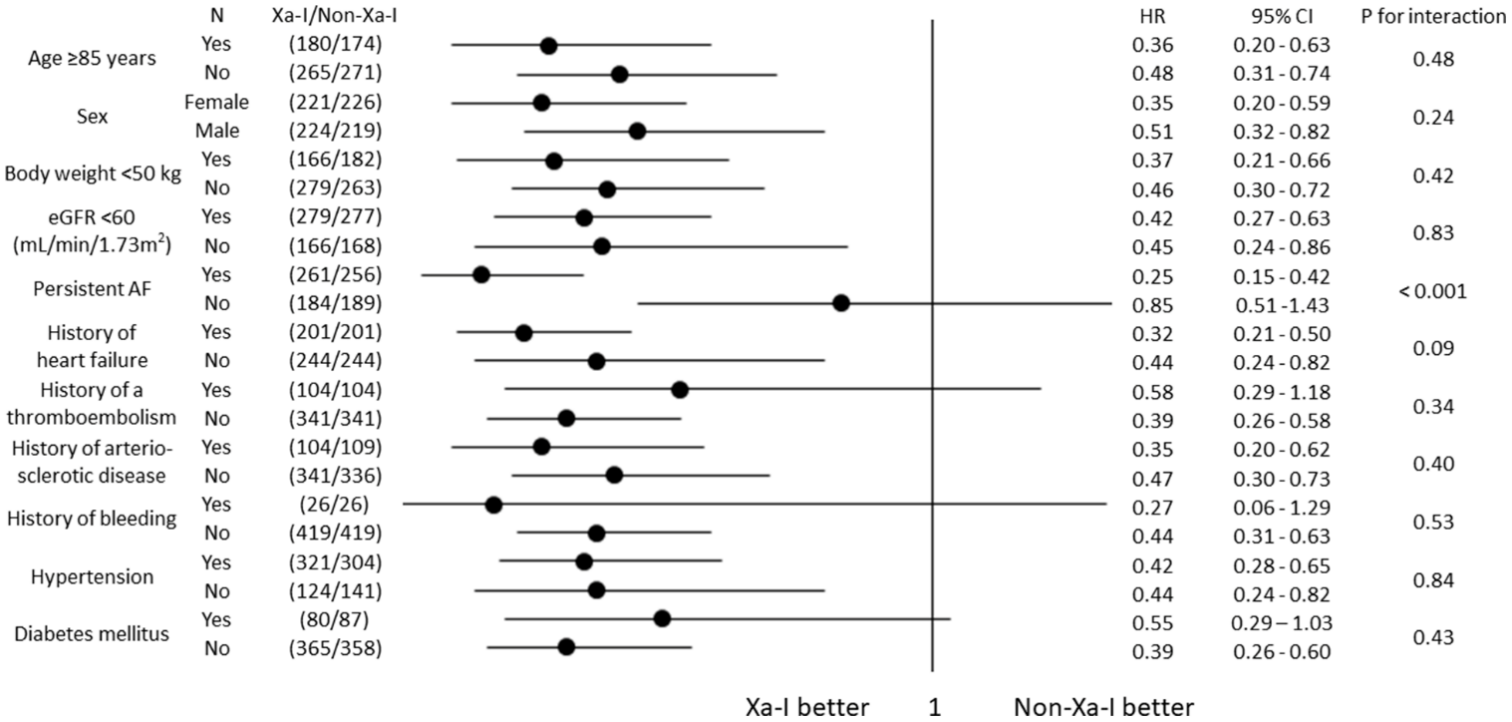
**Subgroup analyses for incidence of cardiovascular events (propensity score-matched cohort).** Subgroup analyses for the incidence of cardiovascular events based on the oral anticoagulant type showed consistent findings. AF, atrial fibrillation; CI, confidence interval; eGFR, estimated glomerular filtration rate; HR, hazard ratio; Xa-I, Factor Xa inhibitor.

The 5-year occurrence of cardiovascular events was significantly lower in the Xa-I group than in the non-Xa-I group in the propensity score-matched cohort (Figure 2A). The secondary outcomes were significantly and numerically lower in the Xa-I group than in the non-Xa-I group (congestive heart failure: hazard ratio [HR] 0.44, 95% confidence interval [CI] 0.29-0.66, P < 0.001; arteriosclerotic disease: HR 0.47, 95% CI 0.22-1.04, P = 0.060; and cardiovascular death: HR 0.41, 95% CI 0.23-0.75, P = 0.003) (Figure 2B–2D). The multivariate Cox proportional hazards model determined that non-Xa-I use was an independent predictor of 5-year cardiovascular events (Table 3). Subgroup analyses of cardiovascular events according to Xa-I use revealed consistent findings, except for the AF type (Figure 3).

**Table 3:** Predictors of cardiovascular events (total original cohort).

	**Univariable**			**Multivariable**		
	**HR**	**95% CI**	**P-value**	**HR**	**95% CI**	**P-value**
Xa-I use	0.59	0.44–0.79	< 0.001	0.44	0.32–0.60	< 0.001
History of a thromboembolism	0.83	0.60–1.16	0.27	0.84	0.60–1.18	0.31
Hypertension	0.87	0.65–1.16	0.34	0.90	0.67–1.22	0.51
Female sex	1.00	0.76–1.32	0.58	0.96	0.71–1.32	0.82
History of major bleeding	1.07	0.58–1.96	0.83	0.93	0.50–1.74	0.82
Statin use	1.31	0.97–1.77	0.08	0.98	0.70–1.35	0.89
Age ≥85 years	1.17	0.88–1.55	0.28	1.15	0.86–1.54	0.36
β-blocker use	1.27	0.97–1.68	0.08	1.23	0.92–1.64	0.16
Persistent AF	1.33	1.00–1.77	0.053	1.30	0.97–1.74	0.08
Body weight <50 kg	1.17	0.89–1.55	0.27	1.28	0.93–1.76	0.13
Diabetes mellitus	1.84	1.36–2.49	< 0.001	1.53	1.11–2.10	0.009
eGFR <60 mL/min/1.73 m^2^	1.65	1.21–2.24	0.001	1.56	1.14–2.13	0.005
History of arteriosclerotic disease	2.38	1.80–3.15	< 0.001	1.98	1.45–2.72	< 0.001
History of heart failure	3.58	2.62–4.90	< 0.001	3.50	2.49–4.91	< 0.001

Abbreviations: AF, atrial fibrillation; CI, confidence interval; eGFR, estimated glomerular filtration rate; HR, hazard ratio; Xa-I, Factor Xa inhibitor.

**Figure 4:**
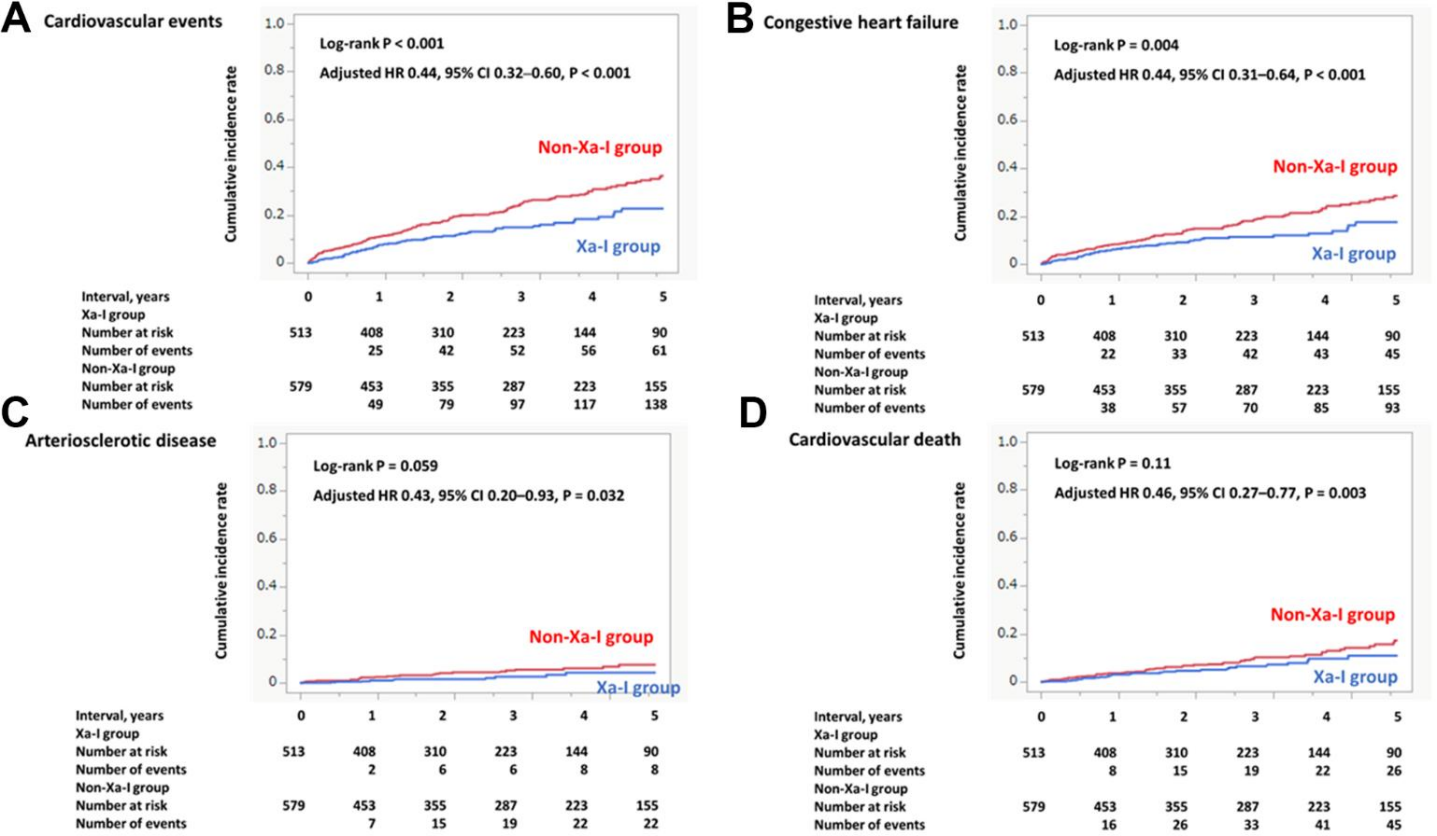
**Incidence of primary and secondary outcomes (total original cohort).** (**A**) Cardiovascular events. (**B**) Congestive heart failure. (**C**) Arteriosclerotic disease. (**D**) Cardiovascular death. The incidence of composite cardiovascular events, arteriosclerotic disease, congestive heart failure, and cardiovascular death within 5 years was significantly lower in the Xa-I group than in the non-Xa-I group. CI, confidence interval; HR, hazard ratio; Xa-I, Factor Xa inhibitor.

The sensitivity analyses of 5-year cardiovascular events, congestive heart failure, arteriosclerotic disease, and cardiovascular death in the total original cohort showed similar results to those of the main analyses (Figure 4A–4D).

As the post-hoc outcomes, the 5-year incidence of strokes was significantly lower in the Xa-I group than in the non-Xa-I group both in the total original cohort and propensity score matched cohort (total original cohort: 7% [n = 36/513] vs. 22% [n = 125/579] P < 0.001; propensity score matched cohort: 7% [n = 33/445] vs. 21% [n = 95/445], P < 0.001). The 5-year incidence of the all-cause death was significantly lower in the Xa-I group than in the non-Xa-I group both in the total original cohort and propensity score matched cohort (total original cohort: 30% [n = 154/513] vs. 47% [n = 270/579], P < 0.001; propensity score matched cohort: 29% [n = 130/445] vs. 58% [n = 260/445], P < 0.001).

An additional analysis comparing the Xa-I and dabigatran groups showed that the 5-year incidence of cardiovascular events and arteriosclerotic disease were significantly lower in the Xa-I group than in the dabigatran group; however, the 5-year incidences of congestive heart failure and cardiovascular death did not differ between the groups (Supplementary Figure 1A–1D).

## DISCUSSION

The present study had several findings. First, the 5-year incidence of cardiovascular events was lower in patients taking Xa-Is than in those taking non-Xa-Is, even after an adjustment for potential confounders. Second, the preventive effects of Xa-Is on congestive heart failure, atherosclerotic disease, and cardiovascular death were confirmed. Third, subgroup analyses exhibited consistent effects of Xa-Is on cardiovascular events across the subgroups, except for the AF type. Fourth, the incidences of cardiovascular events and arteriosclerosis were lower in patients taking Xa-Is than in those taking dabigatran in the total original cohort.

Morimoto et al. suggested some differences in the risk of a myocardial infarction among the OACs in a meta-analysis with an indirect comparison among the OACs [14]. Moreover, a subanalysis of ENGAGE AF-TIMI 48 reported that the reduction in ischemic events, including myocardial infarctions, with edoxaban versus warfarin was greater in patients with coronary artery disease [15]. We clarified the differences between Xa-Is and non-Xa-Is in a long-term observational study among very old patients with NVAF. In the subgroup analyses, the preventive effects of Xa-Is on cardiovascular events were not shown, with dispersed HR in patients with paroxysmal AF. Paroxysmal AF occasionally leads to rapid hemodynamic instability owing to the sudden loss of the atrial function and tachycardias with heart rate vulnerability. A previous study reported that paroxysmal AF, but not persistent AF, was a predictor of hospitalizations for heart failure in patients with heart failure and a reduced ejection fraction [16]. The impact of Xa-Is in patients with paroxysmal AF may differ according to the AF severity or patient condition.

Xa-Is inhibit the Factor Xa-protease-activated receptor 2 (PAR2) activity pathway. Activation of PAR2 causes the progression of cardiac remodeling, the accumulation of inflammatory substances in the myocardium, an increase in interstitial fibrosis, and a reduction in cardiac contractility, leading to heart failure. In fact, a previous study reported that patients receiving Xa-Is exhibited reduced levels of circulating markers of fibrosis and diastolic dysfunction compared with those receiving vitamin K antagonists [10]. The signaling PAR2 activity pathway also induces an increase in vascular inflammatory substances, activates macrophages, and destabilizes atherosclerotic lesions [11]. In fact, experimental studies demonstrated that rivaroxaban inhibits arteriosclerosis development [13, 17]. The mechanism by which dabigatran directly inhibits thrombin differs from that of Xa-Is. Thrombin also contributes to inflammatory responses and fibrosis of vascular smooth muscles, likely related to Factor Xa [18]. However, no experimental study has elucidated the effect of thrombin inhibitors on cardiac remodeling and/or arteriosclerosis. Moreover, a meta-analysis of clinical studies suggested that patients taking dabigatran had a higher incidence of acute coronary syndrome than those taking warfarin [19]. A recent study involving patients with AF and diabetes mellitus who were aged ≥65 years reported a lower incidence of hospitalizations for heart failure in patients taking dabigatran than in those taking warfarin, similar to that in those taking Xa-Is [8]. These results suggest that the effect of dabigatran on the vascular system is limited, but its effect on the cardiac function is expected, supporting our results of the sensitivity analysis regarding dabigatran, with a similar incidence of heart failure and higher incidences of cardiovascular events and arteriosclerotic disease in the dabigatran group than in the Xa-I group in the original total cohort. Inhibition of the activation of the Factor Xa-PAR2 pathway rather than that of coagulation factors may be important. A further study is needed to clarify the association between the Factor Xa-PAR2 pathway and cardiovascular events to enhance the position of Xa-Is beyond only anticoagulant drugs.

To the best of our knowledge, this is the first study to address the effects of Xa-Is on composite cardiovascular events, congestive heart failure, arteriosclerotic disease, and cardiovascular death among very old patients with NVAF over a long-term follow-up period. Our findings could be helpful for the preventive management of cardiovascular diseases, which are becoming increasingly common in the worldwide aging society.

Several limitations of this study should be considered. First, this was a single-center, historical cohort study. However, we could investigate the detailed clinical courses and outcomes owing to the single-center design of the study. We obtained the data from not only the medical records but also the patients, their families, and their primary care physicians. Moreover, the number of patients lost to follow-up during the observation period was small. A high-quality investigation could confirm the reliability of the results of this study. Second, selection bias was inevitable as this study was conducted in an acute referral general hospital managing patients with advanced diseases, and catheter ablation of AF was provided to patients with indications. However, our study provided reliable real-world data in very old patients who did not undergo catheter ablation but were appropriately managed with medication. Third, the much higher rate of all-cause death, especially in the older population, could be a competing risk to the primary and secondary outcomes. Finally, this was a nonrandomized study, and the effect size might be biased with potential confounders. However, the effect of potential confounders would be slight, if any, as all investigated baseline data were adjusted. However, considering that this was a single-center study, the generalizability of our results should be validated in a larger multicenter study.

## CONCLUSIONS

Among patients with NVAF who were aged ≥80 years, the incidence of cardiovascular events, congestive heart failure, arteriosclerotic disease, and cardiovascular death was significantly lower in those taking Xa-Is than in those taking non-Xa-Is during the 5-year observation period. Xa-Is may be useful for not only anticoagulation but also the prevention of cardiovascular events in very old patients with NVAF.

## MATERIALS AND METHODS

### Study design and patients

We conducted a historical cohort study of consecutive patients with NVAF who were aged ≥80 years and received medical treatment for NVAF from March 2011 to February 2021 at Kagawa Prefectural Central Hospital, Kagawa, Japan.

The inclusion criteria were as follows: 1) diagnosis of AF, including a new or existing diagnosis, based on electrocardiogram recordings in the hospital at the time of hospitalization or during outpatient visits; and 2) age ≥80 years at the time of enrollment. The exclusion criteria were as follows: 1) valvular AF; 2) end-stage renal failure; 3) catheter ablation of AF; and 4) nonuse of OACs. Valvular AF was defined as AF with moderate or severe mitral stenosis or a mechanical valve replacement. End-stage renal failure was defined as a creatinine clearance of <15 mL/min/1.73 m2 or regular dialysis.

We determined the first observation day of the study on the basis of the age and OAC use. The first observation day was defined as one of the following: 1) the start date of the study (March 1, 2011) for patients who were aged ≥80 years and were using OACs on that date; 2) the 80th birthday for patients who were aged <80 years and were using OACs on the start date of the study; and 3) the anticoagulation therapy start date for patients who did not use OACs previously.

### Data collection and variables

We collected data on the patient characteristics, including the medical history, laboratory data, medication use, electrocardiography findings, and transthoracic echocardiography findings. The patients were divided into the following two groups based on the inhibitors used: Xa-I group (patients who used the Xa-Is rivaroxaban, apixaban, and edoxaban) and non-Xa-I group (patients who used the non-Xa-Is dabigatran and warfarin). We investigated the outcomes from the medical records at Kagawa Prefectural Central Hospital. We further investigated the outcomes in March 2021 through a mail-in questionnaire or telephone call to patients, their families, and their primary care physicians among patients who completed clinical visits at Kagawa Prefectural Central Hospital. We calculated the time in TTR as an index of warfarin control using Rosendaal’s linear interpolation method [20]. We set the target international normalized ratio at 1.6–2.6 for older patients based on Japanese guidelines [21].

### Study outcomes

The primary outcome was the composite of cardiovascular events. Cardiovascular events included congestive heart failure, arteriosclerotic disease, and cardiovascular death. The secondary outcomes were congestive heart failure, arteriosclerotic disease, and cardiovascular death. Congestive heart failure was defined as heart failure requiring hospitalization. Arteriosclerotic disease included coronary artery disease, peripheral artery disease, and aortic disease. Coronary artery disease included acute coronary syndrome and stable angina requiring interventional treatment (percutaneous coronary intervention and/or coronary artery bypass grafting). Peripheral artery disease was defined as arteriosclerotic obliterans in the lower limbs that required interventional treatment (endovascular and/or surgical therapy). Aortic disease included acute aortic dissection and an aortic aneurysm that required interventional treatment (surgical and/or stent grafting). Cardiovascular death included death caused by congestive heart failure or arteriosclerotic disease, and sudden cardiac death. The post-hoc outcomes were strokes and all-cause death. Strokes included cerebrovascular infarctions, transient ischemic attacks, and intracranial hemorrhages.

### Statistical analysis

We compared the backgrounds and outcomes of the Xa-I and non-Xa-I groups. We censored the observations due to 1) loss to follow-up by the end date of the study (February 28, 2021); 2) a change in or discontinuation of OACs; and 3) catheter ablation of AF. We did not censor cases due to dose changes or drug changes within the group. We performed the main analysis of this study in the propensity score-matched cohort. For the propensity score development, we selected age ≥85 years, sex, body weight <50 kg, estimated glomerular filtration rate (eGFR) <60 mL/min/1.73 m2, hypertension, diabetes mellitus, history of heart failure requiring hospitalization, history of a thromboembolism, history of major bleeding, type of AF, and use of β-blockers as independent variables. We used a logistic regression model to calculate the propensity score with a tolerance of 0.05 and constructed 1:1 matching.

Continuous variables have been expressed as means and standard deviations or medians and IQRs based on their distributions. We used the t-test or Wilcoxon rank-sum test for between-group comparisons. Categorical variables have been presented as numbers and percentages, and intergroup comparisons were conducted using the chi-square test. The outcomes have been presented as the total number of first events. A Kaplan–Meier survival curve and log-rank test were used to compare the incidence rates of the primary and secondary outcomes between the Xa-I and non-Xa-I groups. The effect has been expressed as HR and its 95% CI using a Cox proportional hazards model.

Subgroup analyses were performed in the propensity score-matched cohort to estimate the HRs of cardiovascular events for age, sex, body weight, eGFR, hypertension, diabetes mellitus, history of heart failure requiring hospitalization, history of a thromboembolism, history of arteriosclerotic disease, history of major bleeding, and type of AF.

To assess the robustness of the analyses of the propensity score-matched cohort, we conducted multivariate analyses in the total original cohort. We included the following variables in the multivariate Cox proportional hazards model: age ≥85 years, female sex, body weight ≤50 kg, eGFR <60 mL/min/1.73 m2, hypertension, diabetes mellitus, history of heart failure requiring hospitalization, history of thromboembolism, history of arteriosclerotic disease, history of major bleeding, use of β-blockers, use of statins, and type of AF.

Sensitivity analyses were performed for the primary and secondary outcomes between the Xa-I and non-Xa-I groups on the total original cohort in the same way as the main analyses. Moreover, to evaluate the difference among the types of DOACs, additional analysis was conducted to compare the primary outcome between the Xa-I and dabigatran groups on the total original cohort.

All statistical analyses were performed using SPSS Statistics version 28 software (IBM Corp., Armonk, NY, USA). A two-sided P-value of <0.05 was considered statistically significant.

## Supplementary Material

Supplementary Figure 1
