# Supplementary material for: Impact of Factor Xa inhibitors on cardiovascular events in older patients with nonvalvular atrial fibrillation
Source: Aging (Albany NY). 2025 Apr 10;17(4):982–93. doi: 10.18632/aging.206238 (PMC12074823; doi:10.18632/aging.206238)
Supplement: Supplementary Figure 1 [file aging-17-206238-s001.pdf]

SUPPLEMENTARY FIGURE

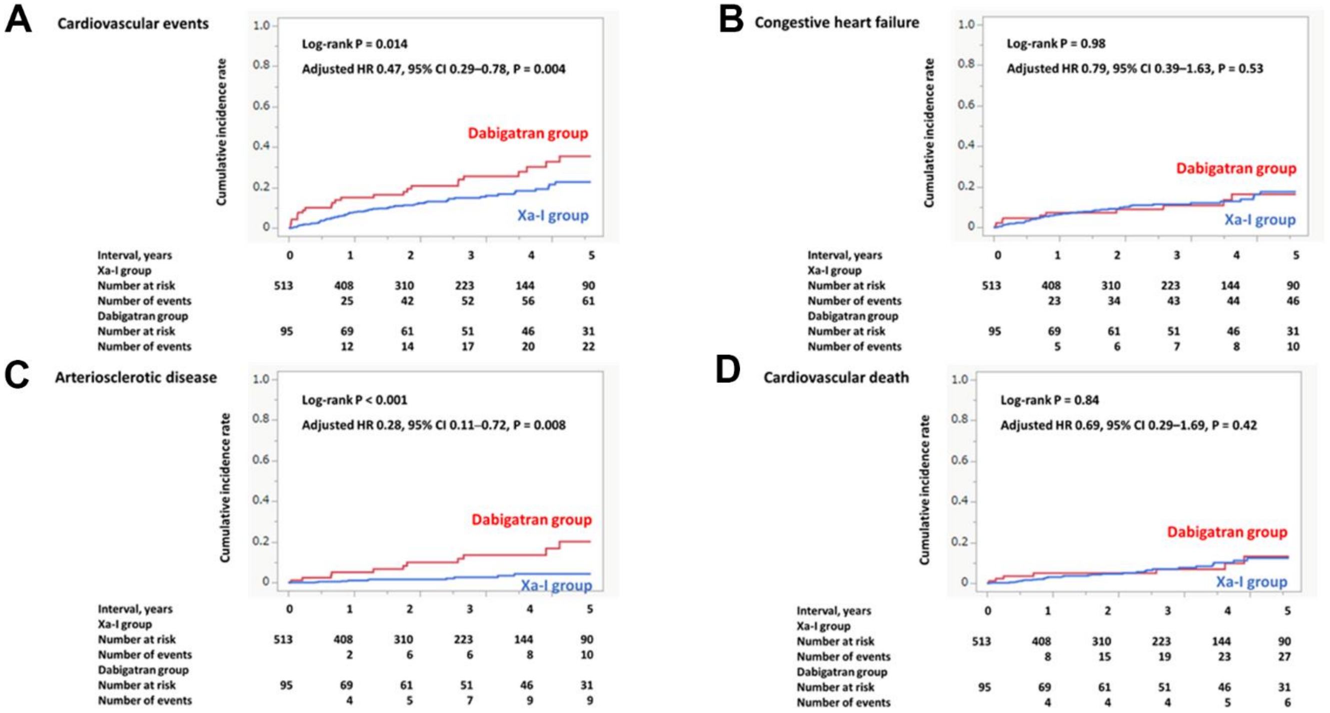

Supplementary Figure 1. Incidence of primary and secondary outcomes: Xa-I vs. dabigatran (total original cohort). The incidence of cardiovascular events and arteriosclerotic disease was significantly lower in the Xa-I group than in the dabigatran group. The incidence of congestive heart failure and cardiovascular death did not differ between the groups. Abbreviations: CI, confidence interval; HR, hazard ratio; Xa-I, Factor Xa inhibitor.
